# Characterization of Resistance Gene Analogues (*RGAs*) in Apple (*Malus* × *domestica* Borkh.) and Their Evolutionary History of the Rosaceae Family

**DOI:** 10.1371/journal.pone.0083844

**Published:** 2014-02-05

**Authors:** Michele Perazzolli, Giulia Malacarne, Angela Baldo, Laura Righetti, Aubrey Bailey, Paolo Fontana, Riccardo Velasco, Mickael Malnoy

**Affiliations:** 1 Research and Innovation Centre, Fondazione Edmund Mach, San Michele all’Adige, Italy; 2 United States Department of Agriculture-Agricultural Research Service Plant Genetic Resources Unit, Geneva, New York, United States of America; Instituto Valenciano de Investigaciones Agrarias, Spain

## Abstract

The family of resistance gene analogues (*RGAs*) with a nucleotide-binding site (NBS) domain accounts for the largest number of disease resistance genes and is one of the largest gene families in plants. We have identified 868 *RGAs* in the genome of the apple (*Malus* × *domestica* Borkh.) cultivar ‘Golden Delicious’. This represents 1.51% of the total number of predicted genes for this cultivar. Several evolutionary features are pronounced in *M. domestica*, including a high fraction (80%) of *RGAs* occurring in clusters. This suggests frequent tandem duplication and ectopic translocation events. Of the identified *RGAs*, 56% are located preferentially on six chromosomes (Chr 2, 7, 8, 10, 11, and 15), and 25% are located on Chr 2. *TIR-NBS* and non*-TIR-NBS* classes of *RGAs* are primarily exclusive of different chromosomes, and 99% of non-*TIR-NBS RGAs* are located on Chr 11. A phylogenetic reconstruction was conducted to study the evolution of *RGAs* in the Rosaceae family. More than 1400 *RGAs* were identified in six species based on their NBS domain, and a neighbor-joining analysis was used to reconstruct the phylogenetic relationships among the protein sequences. Specific phylogenetic clades were found for *RGAs* of *Malus*, *Fragaria*, and *Rosa*, indicating genus-specific evolution of resistance genes. However, strikingly similar *RGAs* were shared in *Malus*, *Pyrus*, and *Prunus*, indicating high conservation of specific *RGAs* and suggesting a monophyletic origin of these three genera.

## Introduction

When a genome sequence is available, the analysis of large gene families can contribute to the understanding of major events responsible for molecular evolution. This is the case for resistance gene analogues (*RGAs*) with a nucleotide-binding site (NBS) domain [1]–[5]. The NBS domain is part of the larger NB-ARC domain that hydrolyses ATP and GTP and functions as a molecular switch for signal transduction after pathogen recognition [6]. Many resistance proteins encoded by *RGAs* contain a leucine-rich repeat (LRR) domain [7], [8], involved in protein–protein interactions and in pathogen recognitions [9]. Proteins codified by *RGAs* can be further classified according to the presence of the toll/interleukin-1 receptor (TIR) or other N-terminal features, such as coiled-coil (CC) and BED finger (Bed) [3], [10], [11]. The N-terminal features are involved in downstream specificity and signaling regulation [12]. *RGAs* evolved for pathogen recognition and frequently matched with specific pathogen avirulence factors to trigger signal transduction cascades and defense responses [9].

The genome sequencing of model plants has enabled the study of *RGA* families in monocots and dicots, including *Arabidopsis thaliana* [11], [13], *Brassica rapa* [14], *Carica papaya* [15], [16], *Cucumis sativus* [17], *Glycine max* [18], [19], *Zea mays* [20], [21], *Medicago truncatula* [22], *Oryza sativa* [23]–[25], *Populus trichocarpa* [26], *Sorghum bicolor* [27], *Vitis vinifera* [2], [5],[28],[29], *Brachypodium distachyon* [30], [31], *Solanum tuberosum* [32], and *Solanum lycopersicum* [33]. According to these studies, approximately 0.2–1.3% of genes predicted in plant genomes corresponds to *RGAs*, which occur at a density of 0.3–1.6 per mega base (Mb). The genome of apple (*Malus* × *domestica* Borkh.) also contains a large number of *RGAs* [34]. Apple is characterized by recent whole genome duplication (WGD) [34]. The role and relevance of such radical genomic changes in plant evolution was largely demonstrated, but the number and timing of WGDs in the different plant species was only partially understood [35], . Polyploidy is common in angiosperms [22], [37], and most if not all extant species are thought to be ancient polyploids [38]. However, ancestral genomes are in most cases dispersed on multiply rearranged chromosomes, having also suffered wholesale gene losses [5], [39]. Given that synonymous substitutions are immune to selection pressure [40], the per-site synonymous substitution rate (Ks) is widely used to infer the time of WGD and to describe the relationships among chromosomes [2], [34].

In this study, cluster organization of *RGAs* and their distribution across chromosomes were analyzed in terms of recent duplication of the apple genome. In addition, the phylogenesis of *RGAs* from the domesticated and wild *Malus* species, including also other Rosaceae, *P. trichocarpa*, and *V. vinifera RGAs*, was considered to clarify the evolutionary history of apple and its related species.

## Results

### Classes of *RGAs* in *Malus* × *domestica*

Based on the presence of the NBS domain, 868 *RGAs* were identified in the genome of the *M. domestica* cultivar ‘Golden Delicious’, and all of them showed a significant (more than 90%) protein similarity with *RGAs* of *A. thaliana*, *P. trichocarpa*, and *V. vinifera*. In addition, 124 putative *RGA* alleles were found, and they were not further analyzed. By domain analysis, *RGAs* were assigned to *TIR-NBS-LRR* (*TNL*) and *CC-NBS-LRR* (*CNL*) classes. In particular, 505 *RGAs* were classified as *NBS-LRR* (*NL*), including *CNL* subclass, and 231 *RGAs* were classified as *TIR-NBS* (*TN*), including *TIR-NBS-LRR* (*TNL)*, *NBS-LRR-TIR* (*NLT*), *TIR-CC-NBS-LRR* (*TCNL*), *TIR-CC-NBS* (*TCN*), and *TIR-NBS* (*TN*) subclasses (Table 1). In addition, 132 *RGAs* were characterized only by the presence of the NBS (N) or CC-NBS (CN) domains.

**Table 1:** Classification and organization of resistance gene analogues (*RGAs*) with a nucleotide-binding site (NBS) domain in different plant genomes.

Characteristic	*Malus ×* *domestica*	*Arabidopsis* *thaliana*	*Populus* *trichocarpa*	*Vitis* *vinifera*	*Oryza* *sativa*	*Cucumis* *sativus*	*Carica* *papaya*	*Sorghum* *bicolor*	*Brassica* *rapa*	*Brachypodium* *distachyon*	*Glycine* *max*	*Zea* *mays*
Number of totalpredicted genes	57,524	27,228	45,654	33,514	41,911(28,236 [30])	26,682	28,591	27,640	nd	25,532	46,430	32,540
Genome size (Mb)	750	125	485	487	389	243	372	730	529	272	1,115	2,500
N° of *RGAs*	868	178	402	391	535	61	54	211(245 [30])	92	178(238 [30])	429 [30]	129 [80]
NBS-LRR class^a^	505	57	236	194	480	nd	31	184	17	212	236	95
	(58%)	(32%)	(59%)	(57%)	(89%)		(57%)	(74%) [30]	(18%)	(89%) [30]	(55%) [30]	(74%) [30]
TIR-NBS class^b^	231	115	94	42	3	nd	7	2	42	nd	154	nd
	(27%)	(64%)	(23%)	(13%)	(1%)		(13%)	(1%) [31]	(46%)		(36%) [30]	
Other *RGAs* c	132	6	72	103	52	nd	16	61	33	27	39	34
	(15%)	(4%)	(18%)	(30%)	(10%)		(30%)	(25%) [30]	(36%)	(11%) [30]	(9%) [30]	(26%) [30]
*RGAs/*totalgenes (%)	1.51	0.65	0.88	1.01	1.27	0.23	0.19	0.76(0.88 [30])	nd	0.69(0.9 [30])	0.92 [30]	0.39 [30]
*RGAs* per Mb	1.16	1.42	0.82	0.7	1.5	0.25	0.15	0.28(0.33 [30])	0.92	0.65(0.87 [30])	0.38 [30]	0.056 [30]
Average numberof exons in *RGAs*	4.51	4.19	2.35	3.96	3.72	nd	nd	nd	4.2	nd	nd	nd
Number of Single *RGAs*	156	46	135	55	104	20	12	nd	18	nd	nd	nd
Number of Clusters	152	39	75	52	157	11	13	nd	24	nd	nd	nd
Maximum Numberof *RGAs* in clusters	21	11	19	26	11	9	7	nd	5	nd		
Average Numberof *RGAs* in cluster	4.11	3.21	3.75	5.78	3.48	3.72	2.92	nd	2.54	nd	nd	nd
Source	this paperand [34]	[11]	[26]	[2], [29]	[24], [25]	[17]	[16]	[27]	[14]	[31]	[19]	[21]

a NBS-LRR class includes: NBS-LRR (NL) and CC-NBS-LRR (CNL). Percentage (%) of this class relative to the total number of *RGAs*is reported in brackets.

b TIR-NBS class includes: TIR-NBS-LRR (TNL), NBS-LRR-TIR (NLT), TIR-CC-NBS-LRR (TCNL), TIR-CC-NBS (TCN), and TIR-NBS (TN). Percentage (%) of this class relative to the total number of *RGAs*is reported in brackets.

c Class of other *RGAs* includes: NBS (N) and CC-NBS (CN). Percentage (%) of this class relative to the total number of *RGAs*is reported in brackets.

nd: not declared by the authors.

The 868 *RGAs* accounted for 1.51% of *M. domestica* predicted genes, a percentage slightly higher than that in other plant genomes (Table 1). The density of *RGAs* per Mb was similar for *M. domestica* and other genomes with the exception of *Z. mays*, *C. papaya*, *C. sativus*, and *S. bicolor*.

The mean exon number detected in apple *RGAs* was 4.51, and the number of exons of *CNL* class (3.46) was lower than the number of *TNL* class (6.41; *P*<0.001). Thus, the number of exons in *RGAs* of *M.* domestica was consistent with the number in *A. thaliana* and *B. rapa* but higher than the number in *V. vinifera, P. trichocarpa*, and *O. sativa* (Table 1). Moreover, 23% of *CNL RGAs* are encoded by a single exon, while all *TNL* have at least three exons.

### Genome Organization and Phylogeny of *RGAs* in *Malus × domestica*

**Figure 1:**
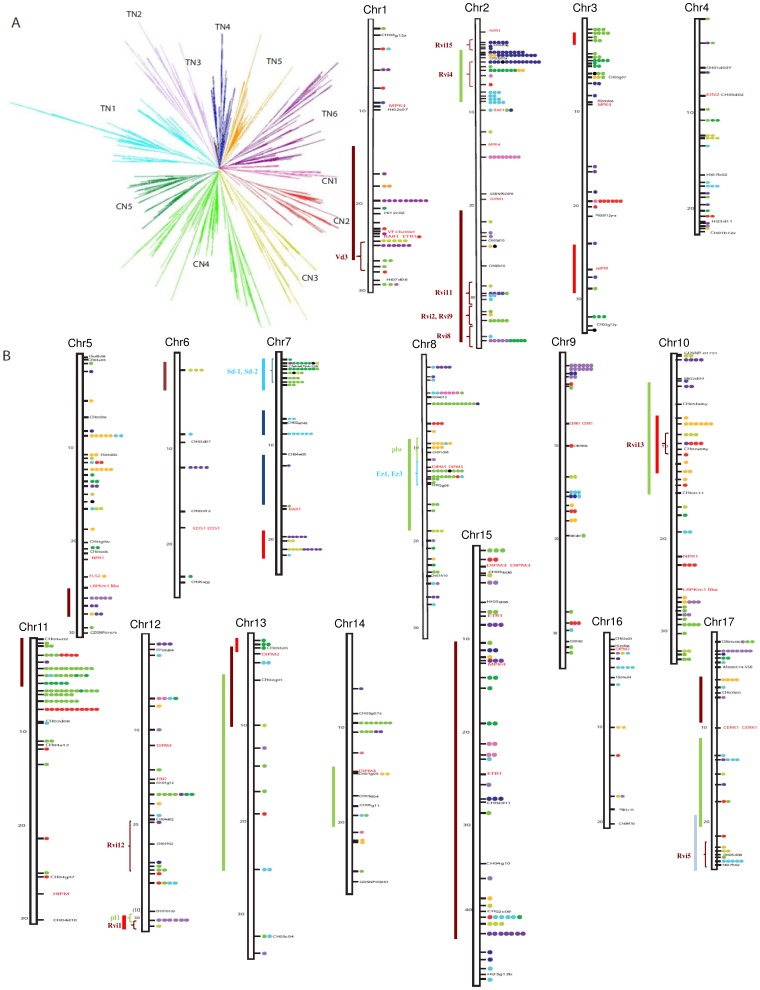
Chromosomal organization of *RGAs* in *Malus × domestica*. **A:** Phylogenetic analysis of NBS domain was carried out by neighbor-joining method [65] on RGAs protein sequences from *M. domestica* cultivar ‘Golden delicious’. Major phylogenetic clades (from CN1 to CN5 and from TN1 to TN6) correspond to the classification based on protein domains. TN1 (light blue): TIR-NBS-LRR; TN2 (light purple): TIR-NBS-LRR and TIR-NBS; TN3 (black): TIR-NBS-LRR; TN4 (blue): TIR-NBS-LRR, CC-TIR-NBS, and TIR-NBS; TN5 (orange): TIR-NBS-LRR, and TIR-NBS; TN6 (dark purple): TIR-NBS-LRR; CN1 (pink): CC-NBS-LRR; CN2 (red): CC-NBS-LRR and NBS-LRR; CN3 (light green): CC-NBS-LRR, NBS-LRR, NBS; CN4 (green): CC-NBS-LRR, NBS-LRR, NBS; CN5 (dark green): CC-NBS-LRR, NBS-LRR, NBS. **B:** *RGAs* assigned to chromosomes (Chr) are represented by dots with colors corresponding to major phylogenetic clades. The size of each chromosome is given in megabase (Mb, on the left side), whereas the markers of the genetic map are shown in black (on the right side). Resistance-related genes different from *RGAs* are shown in red. Known quantitative trait loci (QTL) for resistance to apple scab (brown), powdery mildew (green), aphids (light blue), fire blight (red) and rust mite (blue) are shown by bars on the left side of chromosomes [67]–[73], together with the major resistance genes to apple scab (*Vd3* and *Rvi* genes) [74]–[76], powdery mildew (P*l1*) [77], and aphids (*Sd-1*, *Sd-2*, *Er1*, *Er2*) [78], [79].

Contigs anchored to the genome were used to assess the distribution of *RGAs* in the apple genome [34]. Of the *RGAs*, 778 (90%) were located across the 17 apple chromosomes (Figure 1). Among the anchored *RGAs*, 435 (56%) were assigned to six chromosomes: Chr 2, 7, 8, 10, 11, and 15 (Figure 1 and Table 2). Conversely, Chr 4, 6, 13, 14, and 16 had a low content of *RGAs* (27, 9, 17, 22, and 14 *RGAs*, respectively). *RGAs* were mainly (80%) grouped in clusters, 152 clusters included the majority (622) of the *RGAs* (Figure 1, Table 2 and Table S1). On average, four *RGAs* were present in a cluster, and the largest cluster contained 21 *RGAs* (located on Chr 2). Several clusters of *RGAs* can be associated with QTLs affecting disease resistance of *Malus* genotypes (Figure 1).

**Table 2:** Organization and distribution of resistance gene analogues (*RGAs*) with a nucleotide-binding site (NBS) domain in the apple (*Malus × domestica*) chromosomes.

Chromosome	Number of *RGAs*	Genome organization of *RGAs*		
		Number of single *RGAs*	Number of Clusters	Average Number of *RGAs*/cluster
1	43	10	7	4.7
2	109	14	15	6.3
3	47	12	11	3.2
4	27	12	6	2.5
5	48	11	11	3.4
6	9	2	2	3.5
7	57	4	11	4.8
8	76	11	14	4.6
9	40	7	10	3.3
10	56	14	14	3.0
11	79	7	10	7.2
12	37	11	6	4.3
13	17	9	4	2.0
14	22	6	4	4.0
15	58	14	14	3.1
16	14	3	4	2.8
17	39	9	8	3.8
Not anchored *RGAs*	90	–	–	–
Total	868	156	152	4.1

As previously shown in *Arabidopsis* [6], [11], *RGAs* of *TIR-NBS* and non*-TIR-NBS* classes had different topologies in the phylogenetic analysis (Figure 1A). In particular, six major *TIR-NBS* clades (numbered from TN1 to TN6) and five non-*TIR-NBS* major clades (numbered from CN1 to CN5) were identified in apple. *RGAs* of *TIR-NBS* class were mainly located on Chr 2, 5, 9, 12, 15, 16, and 17, with Chr 16 hosting the *TIR-NBS* class almost exclusively (Figure S1A and Table S1). Chr 3, 4, 8, 11, 13, and 14 were mainly characterized by non-*TIR-NBS* class, and Chr 11 had almost exclusively *RGAs* of non-*TIR-NBS* class. Considering *TIR-NBS* and non*-TIR-NBS* phylogenetic clades, the major clade TN6 represented more than one-third of the *RGAs* on Chr 1 and 6, while the major clade CN4 included more than half of the *RGAs* on Chr 11 and 14 (Figure S1A). Moreover, the major clade TN4 was located preferentially (63%) on Chr 2 (Figure S1B).

### Phylogeny of *RGAs* in Domesticated and Wild *Malus* Species

**Figure 2:**
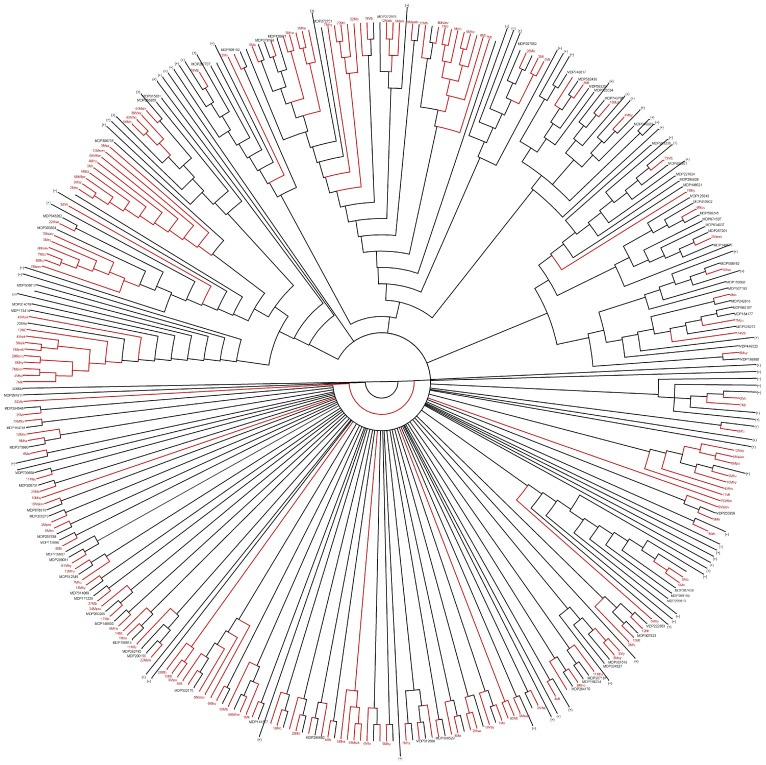
Phylogenesis of *RGAs* from *Malus × domestica* and from wild *Malus* species. Phylogenetic analysis of the NBS domain was carried out by the neighbor-joining method [65] using *RGA* sequences of *M.* domestica cultivar ‘Golden delicious’ (black) and wild *Malus* species (red). Proteins present in contiguous positions on the tree and belonging to the same species are merged (collapsed branches are indicated by the + sign). Phylogentec tree reveals 18 clades specific to *M. domestica*, six clades specific to wild *Malus* species, and 49 clades that include *RGAs* sequences of both domesticated and wild apple species.

Twenty-four wild *Malus* species (Table S2) were considered, and PCR fragments were amplified from germplasm. After sequence comparison, unique fragments were translated in to amino acid sequences (Table S1), and 115 of them matched NBS sequences of known resistance proteins with an E-value lower than 1E^−10^. Phylogenetic analysis indicated that *RGAs* of wild *Malus* species grouped mainly in clades that included sequences of the domesticated apple (Figure 2). A significant fraction of phylogenetic clades contained only a few *RGAs*, probably due to the short sequence of the NBS domain used for this analysis. Some clades consisted mainly of sequences from wild species and contained only few *RGAs* of the domesticated apple.

### Phylogeny of *RGAs* among Rosaceae Species

**Figure 3:**
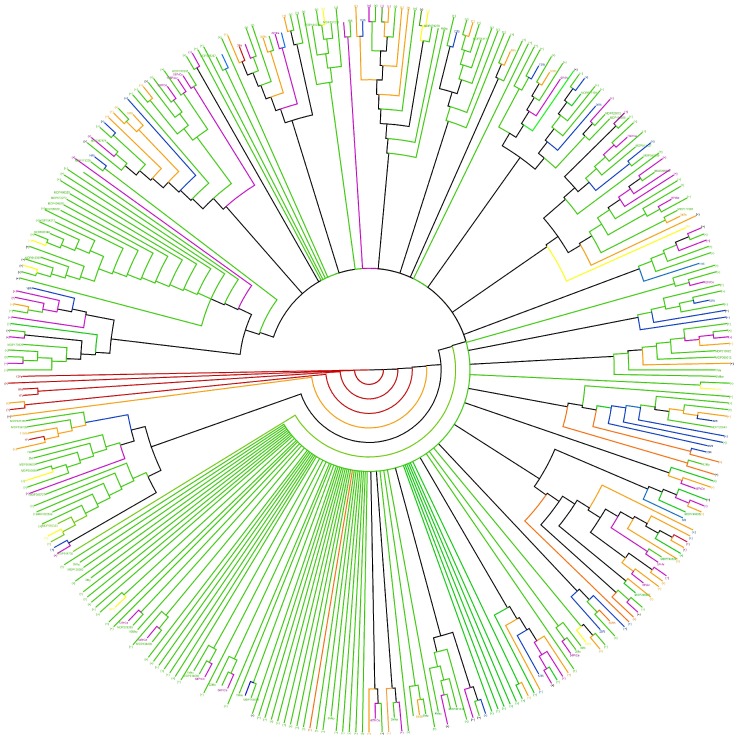
Phylogenesis of *RGAs* from *Malus* species (wild and domesticated apple), *Pyrus communis*, *Prunus* species, *Fragaria ananassa*, *Rubus idaeus*, and *Rosa* species. Phylogenetic analysis of the NBS domain was carried out by the neighbor-joining method [65] using *RGA* sequences of domesticated and wild *Malus* species (green), *Pyrus* spp. (yellow), *Prunus* spp. (purple), *Fragaria* spp. (red), *Rosa* spp. (orange), and *Rubus* spp. (blue). Proteins present in contiguous positions of the tree are merged (collapsed branches are indicated by the + sign). Phylogentec tree indicates 49, three and one clades specific to *Malus* spp., *Fragaria* spp. and *Rosa* spp., respectively. Clades with *RGAs* of different genera: three clades of *Malus* spp. and *Prunus* spp.; seven clades of *Malus* spp. and *Pyrus* spp.; two clades of *Malus* spp. and *Rubus* spp.; four clades of *Malus* spp. and *Rosa* spp.; two clades of *Fragaria* spp. and *Rosa* spp.; two clades of *Malus* spp., *Rosa* spp., and *Rubus* spp.; three clades of *Malus* spp., *Pyrus* spp., and *Rosa* spp.; three clades of *Malus* spp., *Prunus* spp., and *Rubus* spp.; four clades of *Malus* spp., *Prunus* spp., *Rosa* spp., and *Rubus* spp.; three caldes of *Malus* spp., *Prunus* spp., *Pyrus* spp., *Rosa* spp., and *Rubus* spp.; two clades of *Malus* spp., *Fragaria* spp., *Prunus* spp., *Rosa* spp., and *Rubus* spp., one clade of *Malus* spp., *Fragaria* spp., *Pyrus* spp., *Rosa* spp., and *Rubus* spp.

A total of 693 Rosaceae *RGA* sequences at NCBI were downloaded (75 from *Rubus*, 293 from *Prunus*, 16 from *Fragaria*, 125 from *Rosa*, 34 from *Pyrus*, and 150 public sequences from *Malus* species) and compared to the 868 *RGAs* of *M. domestica* and the 210 sequences obtained from wild *Malus* species (Table S1). In the phylogenetic tree of Rosaceae species (Figure 3), 49 clades were specific to the genus *Malus*, and included sequences from two or more *Malus* species. Most of the remaining clades were represented by *RGAs* from two or more Rosaceae genera. In particular, three clades comprised *RGAs* of *Malus*, *Pyrus*, and *Prunus*, indicating a monophyletic origin of the three genera and strong conservation of some *RGA* sequences in these plants. Few clades were represented by non-apple *RGAs*, and clades specific to *Fragaria* or *Rosa* were also present.

### Comparison of *RGAs* among *Malus × domestica*, *Populus trichocarpa*, and *Vitis vinifera*

**Figure 4:**
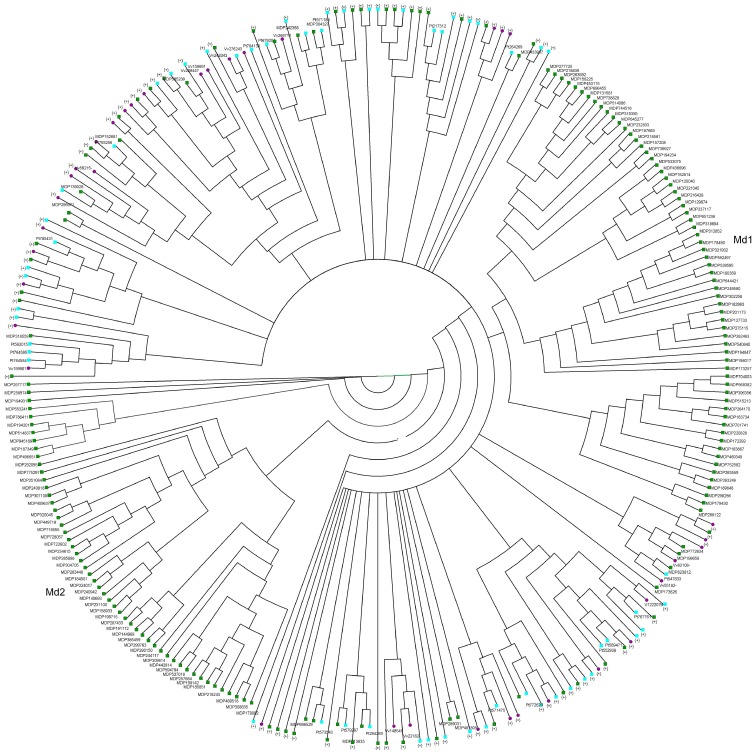
Phylogenesis of *RGAs* from *Malus* species (wild and domesticated apple), *Populus trichocarpa* and *Vitis vinifera*. Phylogenetic analysis of the NBS domain was carried out by the neighbor-joining method [65] using *RGA* sequences of domesticated and wild *Malus* species (green), *P. trichocarpa* (cyan), and *V. vinifera* (purple). Proteins present in contiguous positions on the tree and belonging to the same species are merged (collapsed branches are indicated by the + sign). Two phylogenetic clades comprise only sequences of *M. domestica* (Md1 and Md2).

*RGA* sequences can also be compared across different plant families, and a phylogenetic tree of *RGAs* from *M. domestica*, wild *Malus* species, *V. vinifera*, and *P. trichocarpa* (Table S1) was obtained (Figure 4). Several clades included sequences from two or three species, and two major clades, named Md1 and Md2, comprised only sequences of *M. domestica* (Figure 4). However, sequences of the Md1 clade were grouped in three subclades in the phylogenetic tree of *RGAs* from Rosaceae species (Figure S2). *RGAs* of subclades Md1 sc2 and Md1 sc3 did not show similarity with any Rosaceae *RGAs,* whereas sequences of Md1 subclade 1 (Md1 sc1) shared significant similarity with four *RGAs* of *Pyrus* (Figure S2). Clade Md2 included one and two *RGAs* from *Rubus* and *Rosa*, respectively. Most of the *RGAs* of the clade Md2 are located on Chr 2, 3, 7, 11, 12, and 15.

### Duplication of *RGAs* in *Malus × Domestica*

To study the recent duplication of *RGAs* in the *M. domestica* genome, Ks values were determined, and results from recent gene duplications were highlighted (Figure S3). Links among different *RGAs* helped to describe the relationships among the duplicated apple chromosomes [34]. Homologous apple chromosomes had more than 10 links, except for Chr 13 and 16, which hosted only a low number of *RGAs*. Chr 6 was not included in this analysis because it contains only nine *RGAs*, six of them derived from the recent WGD. Moreover, the duplicated chromosomes had *RGAs* belonging to the same phylogenetic clades (Figure S4).

## Discussion

To counteract pathogens, plants rely on the innate immunity of their cells and on systemic signals emanating from infection sites [9], [41]. Pathogen effectors from very diverse organisms are recognized by resistance proteins encoded by *RGAs* and activate plant defense responses [6], [9]. NBS-mediated disease resistance is effective against obligate biotrophic and hemibiotrophic pathogens but not against necrotrophs, which kill host tissues during colonization [42].

In apple, the abundance of *RGAs* is only partly related to genome size (750 Mb), which is much smaller than in maize (2300 Mb; [21]) or soybean (1115 Mb; [19]). The *TIR-NBS* class accounts for the largest group of *RGAs* in *A. thaliana* (64%; [11]) and *B. rapa* (64%; [14]). In *P. trichocarpa* [26], *V. vinifera* [2], [5], [28], [29], and *C. papaya* [16], [30], the percentage of *TIR-NBS* class is much lower than in the previously mentioned species. The *TIR-NBS* class is present at a very low frequency in *O. sativa* (1%; [24]) and *S. bicolor* (1%; [27]) and is absent in *B. distachyon* and *Z. mays* [30], supporting the conclusion that this class is specific for dicotyledons. In apple, 231 *RGAs* of *TIR-NBS* class have been identified, and they are mainly located on Chr 2, 5, 9, 12, 15, 16, and 17. However, the number of *RGAs* belonging to non-*TIR-NBS* class in apple (505) is greater than in all other species considered, and these *RGAs* are mainly located on Chr 3, 4, 8, 11, 13, and 14. The existence of chromosome-specific *RGAs* classes suggests that groups of chromosomes evolved separately, but further analyses are required to test this hypothesis. In grapevine, the existence of two chromosome groups has been inferred based on *RGAs* cluster similarity, and the two groups seem to have evolved independently [2]. Moreover, the TIR-NBS class is specific for only one of the two components of *V. vinifera* genome, suggesting an independent evolution of the *RGA* classes [2].

In apple, 56% of *RGAs* (435 of 778 anchored) are located preferentially on six chromosomes, with 14% located on Chr 2. In large gene families, genes are commonly organized in clusters and superclusters [4], [5], [11], [14], [16], [25], [26], as demonstrated here for the apple genome. Of the *RGAs* clusters in apple, 71% (108 of 152) include *RGAs* from the same phylogenetic clade, and 29% *RGAs* from two to three different clades. Clusters frequently consist of tandem duplications of the same gene [5], [43]. Heterogeneous clusters, in which sequences belong to different phylogenetic lineages, are also present, most probably as a result of different molecular mechanisms like ectopic recombination, chromosomal translocation, and gene transposition, as has been recently highlighted for the grapevine genome [2]. This kind of genome evolution could be explained in terms of a positive selection for cluster complexity, which could serve as the basis for the generation of new resistance specificities [4], [44]. The role of tandem duplication in the apple genome is supported by low Ks values among *RGAs* of the same cluster, as is already known for other species [2], [5], [14], [22], [43]. Gene duplication in a position different from the original cluster has to be preceded by gene transposition, as predicted for *A. thaliana* and *V. vinifera RGAs* [1], [2]. Thus, a successful transposition is the starting point for the creation of a new *RGA* cluster, and the selection for disease resistance could favor the process [5], [45]. Moreover, analysis of *RGA* transposition has indicated that *V. vinifera* putative component genomes may have evolved independently and later fused and evolved together in the same nucleus [2].

Velasco *et al.* [34] have shown that recent WGD has increased the chromosome number in apple from nine in the putative ancestor to the current 17. The recent duplication of *RGAs* due to a WGD event supports the existence of i) a tetraploid state of the genome in which a pair of chromosomes exists with a second homologous pair; ii) duplications inside chromosomes, particularly for Chr 11 where recent duplications can be observed; and iii) duplications in different chromosomes, suggesting recent events of gene transposition. Eight of the 17 chromosomes (Chr 3 and 11, 5 and 10, 9 and 17, and 13 and 16) represent a direct duplication of four ancestral chromosomes, and each of the extant Chr 4, 6, 12, and 14 derives from translocation between two ancestral chromosomes [34]. More complex events have generated the remaining five chromosomes that are derived from starting three ancestral chromosomes. The different clades of *RGAs* along duplicated chromosomes indicate a similar position of orthologous *RGAs* along each chromosome doublets (Chr 3 and 11, 5 and 10, 9 and 17, and 13 and 16). These results strongly support the origin of the apple chromosomes as described by Velasco *et al.* [34] and indicate that *RGA* distribution might be used to dissect plant genome evolution [2]. As is the case for other species, the process of gene duplication has shaped the apple genome in different ways, including the selective retention of paralogs associated with specific biological processes, the amplification of specific gene families, and an extensive subfunctionalization of paralogs. Both the major WGD event and small-scale duplications could be responsible for the high number of the apple *RGAs*. A remarkable feature of gene duplication in apple is the high proportion of paralogs showing divergent expression patterns [46]. Extensive subfunctionalization could have contributed to the acquisition of new traits specific to apple or to the Pyrinae lineage [47]. Sequences of Eurosid genomes provide evidence of ancient genome duplications that occurred early in evolution, suggesting a polyploid origin for most Eudicots [28], [48].

Most of the *RGAs* of wild *Malus* species are closely related *RGAs* of the domesticated apple. Whereas *RGAs* sequencing from wild *Malus* species was partial and could include alleles of the same gene, phylogenetic analysis revealed specific clades of wild *Malus* species, indicating, as expected, the potential to enlarge the the genetic variation of *RGAs* in domesticated apple. Moreover, the comparison of apple *RGAs* with those of other Rosaceae indicates the existence of specific clades for apple. In addition, several clades include a mixture of *RGAs* from *Malus*, *Pyrus*, and *Prunus*, indicating that similar resistance genes are still shared in different genera of the Rosaceae. While these results support the monophyletic origin of the three genera, clades specific for each genus were also found. The existence of genus- or species-specific clades indicates the existence of mechanisms for cluster conservation, as reported by Plocik *et al.* [49].

Phylogenetic relationships within the Rosaceae inferred from *RGAs* are consistent with phylogenies based on chloroplast and other nuclear genes [50], [51]. The phylogenetic analysis of the *RGAs* from *Malus*, *Vitis*, and *Populus* shows that *Malus* contains two large non-*TIR-NBS* clades that are specific to *Malus*. This inference should be considered with caution, because the *RGA* sequences used in our analysis are from only a few species. Several other reasons could explain the variation of *RGAs* in Rosaceae species, such as the inter-specific variation of the *RGA* family size observed in dicotyledonous plants. Similar situations were reported for other gene families in the *Archeae* [52], bacteria [52], [53], and mammals [54], [55]. The variation of *RGA* family size between species could be attributed to gene duplication, deletion, pseudogenization, and functional diversification [56]–[58]. The last case is supported by the necessity of a species to adapt to rapidly changing pathogen populations.

### Concluding Remarks

This paper analyses the *RGAs* of *Malus* spp. and other Rosaceae species to reveal specific evolutionary features of *M. domestica*. *RGAs* of *M. domestica* are mainly located in clusters and are mapped preferentially on six chromosomes. *TIR-NBS* and non-*TIR-NBS* classes of *RGAs* are located in different chromosome groups. Phylogenetic reconstruction in the Rosaceae family revealed specific clades of *RGAs* for *Malus* spp., *Fragaria* spp., and *Rosa* spp., indicating genus-specific evolution of resistance genes. However, strikingly similar *RGAs* were shared in different species of *Malus*, *Pyrus*, and *Prunus* highlighting a monophyletic origin of these three genera and the high conservation of some *RGA* sequences in these plants.

## Materials and Methods

### Identification of *RGAs* in the Apple Genome

The RGA sequences were identified from the predicted proteins of *M. domestica* cultivar ‘Golden Delicious’ [34] based on their NB-ARC domain profile (PF00931 [59]) using HMMER [60]. Putative *RGA* alleles were identified as predicted genes that have more than 90% of sequence similarity and overlap with another *RGA* along each scaffold of the heterozygous apple genome. Apple *RGAs* were validated by BLAST-N analysis (more than 90% protein sequence similarity) against known *A. thaliana*, *P. trichocarpa*, and *V. vinifera* genes. *RGAs* were grouped in different classes based on the presence of the domains TIR, LRR, CC, and BED finger [43]. The motifs were derived from the domain profiles retrieved from PFAM (http://pfam.janelia.org), PANTHER (http://www.pantherdb.org/), and SMART (http://smart.embl-heildelberg.de) databases and from the COILS program; a stringent threshold of 0.9 was used so that CC domains were specifically detected [61]. Resistance-related proteins were also identified based on kinase domains (IPR000719, PF07714, PF00069). Additional putative apple resistance genes were selected using BLAST and *Arabidopsis* proteins as reference sequences, based on a 60% similarity threshold.

### Identification of *RGA* Clusters in the Apple Genome

The *Arabidopsis* definition of *RGA* cluster [4] was adopted: two or more *RGAs* in a cluster should be located within an average of 250 Kb and should not be interrupted by more than 21 open reading frames different from *RGAs*, as previously adopted for grapevine *RGA* clusters [2].

### Isolation of *RGAs* from Wild Species

Four pairs of degenerate primers targeting the NBS domain [62], [63] were used to amplify *RGA* sequences from 26 different *Malus* accessions present in the USDA apple germplasm collection at Geneva (NY, USA) (www.ars-grin.gov/npgs/index.html; Table S2). The homologous sequences represent the following species: *M. baccata, M. florentina, M. floribunda, M. fusca, M. halliana, M. honanensis, M. hupehemsis, M. kansuensis, M. micromalus, M. orientalis, M. prattii, M. prunifolia, M. pumila, M. robusta, M. sargentii, M. sieboldii, M. sieversii, M. sikkimensis, M. sublobata, M. sylvestris, M. transitoria,* and *M. yunnanensis* (Table S2). PCR fragments were cloned in pGEMT easy (Promega), and two clones for each fragment were sequenced. Sequences were screened, cleaned, and compared with resistance genes previously identified in Rosaceae and in other Angiosperms. BLAST DNA similarity searches were performed against the *RGA* sequences of the apple genome using a collection of established *RGAs*. The *RGAs* were translated using tBLAST-N. Clones were filtered based on hit quality, because most of the *RGA* clones encoded between 24 and 40 amino acid residues. Queries having only a single hit below 90% identity were removed, and those with multiple smaller hits were annotated manually. *RGA* sequences from wild *Malus* species were submitted to the NCBI database (www.ncbi.nlm.nih.gov) under the accession numbers reported in Table S1.

### Phylogenetic Analyses

Public *RGA* sequences from Rosaceae, *P. trichocarpa*, and *V. vinifera* Release 2 were downloaded from GenBank (http://www.ncbi.nlm.nih.gov; Table S1). *RGA* sequences from wild *Malus* species were also included (Table S1). Protein sequences of NBS domain of *RGAs* from *M. domestica* were aligned together with NBS sequences of wild *Malus* species, *P. trichocarpa*, *V. vinifera* and with the other Rosaceae species using hidden Markov models with the Sequence Alignment and Modeling Software System (SAM-T2K [64]); the sequences were formatted for analysis with the Phylip phylogenetic inference package [65].

The SEQBOOT tool of the Phylip package was used to generate 500 bootstraps of the data set, and the PROTDIST tool was used to construct 500 bootstrapping distance matrices using the Dayhoff PAM matrix [65]. These matrices were jumbled twice and processed with the FITCH tool to create a phylogenetic tree. A neighbor-joining tree of the 500 bootstraps was also constructed (jumbling the sequence input order twice), and a majority-rule consensus tree was assembled.

### Determination of the Ks Value

Based on a CLUSTALW nucleotide alignment of *M. domestica RGAs* sequences, a total of 302253 Ks values were obtained [66]. The connections between chromosomes were defined on the basis of the number of *RGAs* and Ks values. A connection between two chromosomes was accepted if at least ten *RGAs* had a Ks value lower than or equal to the first quartile of 0.25 [34].

## Supporting Information

Figure S1 **A:** Distribution (percentage) of the major phylogenetic clades of apple *RGAs* (Figure1A) on the 17 *M. domestica* chromosomes (Chr). **B:** Percentage of chromosome (Chr) assignment to the major phylogenetic clades. Colours of major phylogenetic clades and chromosomes are listed below each chart.
(TIF)

Figure S2 **Phylogenesis of** ***RGAs*** **from Rosaceae species.** Phylogenetic analysis of the NBS domain was carried out by the neighbor-joining method [65] using *RGA* sequences of domesticated and wild *Malus* species (green), *Pyrus* spp. (yellow), *Prunus* spp. (purple), *Fragaria* spp. (red), *Rosa* spp. (orange), and *Rubus* spp. (blue). The composition of the phylogenetic clades (Md1 and Md2; Figure 4) and subclades (sc) of sequences mainly from *M. domestica* is highlighted. Proteins present in contiguous positions on the tree are merged (collapsed branches are indicated by the + sign).
(TIF)

Figure S3 **Connections between apple chromosomes based on Ks values from pairwise comparisons of** ***RGAs*** **.** Joining lines represent connections between two *RGAs* among duplicated chromosomes [35] (blue, red, pink, green), among not duplicated chromosomes (yellow), and within the same chromosome (gray). Each line represents a connection between two *RGAs* with a Ks value lower than 0.25 [35]. A connection between two chromosomes was accepted if at least ten pairwise comparisons had a Ks value lower than 0.25.
(TIF)

Figure S4 **Distribution of** ***RGAs*** **among chromosome (Chr) doublets derived from the recent whole genome duplication of apple** [**34**] **.** Colours of major phylogenetic clades (Figure 1A) are indicated.
(TIF)

Table S1 **List of accession numbers and abbreviations of resistance gene analogues (** ***RGAs*** **) with a nucleotide-binding site (NBS) domain from** ***Malus × domestica*** **,** ***Populus trichocarpa*** **,** ***Vitis vinifera*** **, wild** ***Malus*** **species,** ***Fragaria*** **spp.,** ***Prunus*** **spp.,** ***Pyrus communis*** **,** ***Rubus idaeus*** **, and** ***Rosa roxburghii.*** Chromosome location, code, and class based on protein domain analysis are indicated for each of the *RGAs* of *M. domestica*.
(XLS)

Table S2 **List of wild** ***Malus*** **species accessions (USDA apple germplasm collection at Geneva, NY, USA;** www.ars-grin.gov/npgs/index.html **) used for the isolation of** ***RGAs*** **.**
(DOCX)

## References

[1] Freeling M, Lyons E, Pedersen B, Alam M, Ming R, et al (2008) Many or most genes in *Arabidopsis* transposed after the origin of the order Brassicales. Genome Res 18: 1924–1937. 18836034 10.1101/gr.081026.108 PMC2593585

[2] Malacarne G, Perazzolli M, Cestaro A, Sterck L, Fontana P, et al (2012) Deconstruction of the (paleo)polyploid grapevine genome based on the analysis of transposition events involving *NBS* resistance genes. PloS one 7: e29762. 22253773 10.1371/journal.pone.0029762 PMC3256180

[3] Meyers BC, Kaushik S, Nandety RS (2005) Evolving disease resistance genes. Curr Opin Plant Biol 8: 129–134. 15752991 10.1016/j.pbi.2005.01.002

[4] Richly E, Kurth J, Leister D (2002) Mode of amplification and reorganization of resistance genes during recent *Arabidopsis thaliana* evolution. Mol Biol Evol 19: 76–84. 11752192 10.1093/oxfordjournals.molbev.a003984

[5] Yang S, Zhang X, Yue JX, Tian D, Chen JQ (2008) Recent duplications dominate NBS-encoding gene expansion in two woody species. Mol Genet Genomics 280: 187–198. 18563445 10.1007/s00438-008-0355-0

[6] Meyers BC, Dickerman AW, Michelmore RW, Sivaramakrishnan S, Sobral BW, et al (1999) Plant disease resistance genes encode members of an ancient and diverse protein family within the nucleotide-binding superfamily. Plant J 20: 317–332. 10571892 10.1046/j.1365-313x.1999.t01-1-00606.x

[7] Leister D, Ballvora A, Salamini F, Gebhardt C (1996) A PCR-based approach for isolating pathogen resistance genes from potato with potential for wide application in plants. Nat Genet 14: 421–429. 8944022 10.1038/ng1296-421

[8] Yu YG, Buss GR, Maroof MA (1996) Isolation of a superfamily of candidate disease-resistance genes in soybean based on a conserved nucleotide-binding site. Proc Natl Acad Sci U S A 93: 11751–11756. 8876209 10.1073/pnas.93.21.11751 PMC38130

[9] Dangl JL, Jones JDG (2001) Plant pathogens and integrated defence responses to infection. Nature 411: 826–833. 11459065 10.1038/35081161

[10] Bai J, Pennill LA, Ning J, Lee SW, Ramalingam J, et al (2002) Diversity in nucleotide binding site-leucine-rich repeat genes in cereals. Genome Res 12: 1871–1884. 12466291 10.1101/gr.454902 PMC187567

[11] Meyers BC, Kozik A, Griego A, Kuang H, Michelmore RW (2003) Genome-wide analysis of NBS-LRR-encoding genes in Arabidopsis. Plant Cell 15: 809–834. 12671079 10.1105/tpc.009308 PMC152331

[12] DeYoung BJ, Innes RW (2006) Plant NBS-LRR proteins in pathogen sensing and host defense. Nat Immunol 7: 1243–1249. 17110940 10.1038/ni1410 PMC1973153

[13] Tan X, Meyers BC, Kozik A, Al West M, Morgante M, et al (2007) Global expression analysis of nucleotide binding site-leucine rich repeat-encoding and related genes in Arabidopsis. BMC Plant Biol 7: 56. 17956627 10.1186/1471-2229-7-56 PMC2175511

[14] Mun J-H, Yu H-J, Park S, Park B-S (2009) Genome-wide identification of NBS-encoding resistance genes in *Brassica rapa* . Mol Genet Genomics 282: 617–631. 19838736 10.1007/s00438-009-0492-0 PMC2777221

[15] Ming R, Hou SB, Feng Y, Yu QY, Dionne-Laporte A, et al (2008) The draft genome of the transgenic tropical fruit tree papaya (*Carica papaya* Linnaeus). Nature 452: 991–996. 18432245 10.1038/nature06856 PMC2836516

[16] Porter BW, Paidi M, Ming R, Alam M, Nishijima WT, et al (2009) Genome-wide analysis of *Carica papaya* reveals a small *NBS* resistance gene family. Mol Genet Genomics 281: 609–626. 19263082 10.1007/s00438-009-0434-x

[17] Huang S, Li R, Zhang Z, Li L, Gu X, et al (2009) The genome of the cucumber, *Cucumis sativus* L. Nat Genet. 41: 1275–1281. 10.1038/ng.475 19881527

[18] Kang YJ, Kim KH, Shim S, Yoon MY, Sun S, et al. (2012) Genome-wide mapping of *NBS-LRR* genes and their association with disease resistance in soybean. BMC Plant Biol 12. 10.1186/1471-2229-12-139 PMC3493331 22877146

[19] Schmutz J, Cannon SB, Schlueter J, Ma J, Mitros T, et al (2010) Genome sequence of the palaeopolyploid soybean. Nature 463: 178–183. 20075913 10.1038/nature08670

[20] Cheng Y, Li X, Jiang H, Ma W, Miao W, et al (2012) Systematic analysis and comparison of nucleotide-binding site disease resistance genes in maize. Febs J 279: 2431–2443. 22564701 10.1111/j.1742-4658.2012.08621.x

[21] Schnable PS, Ware D, Fulton RS, Stein JC, Wei F, et al (2009) The B73 maize genome: complexity, diversity, and dynamics. Science 326: 1112–1115. 19965430 10.1126/science.1178534

[22] Ameline-Torregrosa C, Wang B-B, O’Bleness MS, Deshpande S, Zhu H, et al (2008) Identification and characterization of nucleotide-binding site-Leucine-rich repeat genes in the model plant *Medicago truncatula* . Plant Physiol 146: 5–21. 17981990 10.1104/pp.107.104588 PMC2230567

[23] Matsumoto T, Wu JZ, Kanamori H, Katayose Y, Fujisawa M, et al (2005) The map-based sequence of the rice genome. Nature 436: 793–800. 16100779 10.1038/nature03895

[24] Monosi B, Wisser RJ, Pennill L, Hulbert SH (2004) Full-genome analysis of resistance gene homologues in rice. Theor Appl Genet 109: 1434–1447. 15309302 10.1007/s00122-004-1758-x

[25] Zhou T, Wang Y, Chen JQ, Araki H, Jing Z, et al (2004) Genome-wide identification of *NBS* genes in *japonica* rice reveals significant expansion of divergent non-*TIR-NBS-LRR* genes. Mol Genet Genomics 271: 402–415. 15014983 10.1007/s00438-004-0990-z

[26] Kohler A, Rinaldi C, Duplessis S, Baucher M, Geelen D, et al (2008) Genome-wide identification of *NBS* resistance genes in *Populus trichocarpa* . Plant Mol Biol 66: 619–636. 18247136 10.1007/s11103-008-9293-9

[27] Paterson AH, Bowers JE, Bruggmann R, Dubchak I, Grimwood J, et al (2009) The *Sorghum bicolor* genome and the diversification of grasses. Nature 457: 551–556. 19189423 10.1038/nature07723

[28] Jaillon O, Aury J-M, Noel B, Policriti A, Clepet C, et al (2007) The grapevine genome sequence suggests ancestral hexaploidization in major angiosperm phyla. Nature 449: 463–U465. 17721507 10.1038/nature06148

[29] Velasco R, Zharkikh A, Troggio M, Cartwright DA, Cestaro A, et al (2007) A high quality draft consensus sequence of the genome of a heterozygous grapevine variety. PloS one 2: e1326. 18094749 10.1371/journal.pone.0001326 PMC2147077

[30] Li J, Ding J, Zhang W, Zhang Y, Tang P, et al (2010) Unique evolutionary pattern of numbers of gramineous *NBS–LRR* genes. Mol Genet Genomics 283: 427–438. 20217430 10.1007/s00438-010-0527-6

[31] Vogel JP, Garvin DF, Mockler TC, Schmutz J, Rokhsar D, et al (2010) Genome sequencing and analysis of the model grass *Brachypodium distachyon* . Nature 463: 763–768. 20148030 10.1038/nature08747

[32] Xu X, Pan S, Cheng S, Zhang B, Mu D, et al (2011) Genome sequence and analysis of the tuber crop potato. Nature 475: 189–194. 21743474 10.1038/nature10158

[33] Andolfo G, Sanseverino W, Rombauts S, Van de Peer Y, Bradeen JM, et al (2013) Overview of tomato (*Solanum lycopersicum*) candidate pathogen recognition genes reveals important Solanum *R* locus dynamics. New Phytologist 197: 223–237. 23163550 10.1111/j.1469-8137.2012.04380.x

[34] Velasco R, Zharkikh A, Affourtit J, Dhingra A, Cestaro A, et al (2010) The genome of the domesticated apple (*Malus × domestica* Borkh.). Nat Genet 42: 833–839. 20802477 10.1038/ng.654

[35] Van de Peer Y, Fawcett JA, Proost S, Sterck L, Vandepoele K (2009) The flowering world: a tale of duplications. Trends Plant Sci 14: 680–688. 19818673 10.1016/j.tplants.2009.09.001

[36] Murat F, Van de Peer Y, Salse J (2012) Decoding plant and animal genome plasticity from differential paleo-evolutionary patterns and processes. Genome Biol Evol 4: 917–928. 22833223 10.1093/gbe/evs066 PMC3516226

[37] Soltis DE, Soltis PS (1999) Polyploidy: recurrent formation and genome evolution. Trends Ecol Evol 14: 348–352. 10441308 10.1016/s0169-5347(99)01638-9

[38] Otto SP, Whitton J (2000) Polyploid incidence and evolution. Annu Rev Genet 34: 401–437. 11092833 10.1146/annurev.genet.34.1.401

[39] Bowers JE, Chapman BA, Rong JK, Paterson AH (2003) Unravelling angiosperm genome evolution by phylogenetic analysis of chromosomal duplication events. Nature 422: 433–438. 12660784 10.1038/nature01521

[40] Li WH, Grauer D (1991) Fundamentals of molecular evolution. Sunderland, MA: Sinauer Associate, Inc. 481 p.

[41] Chisholm ST, Coaker G, Day B, Staskawicz BJ (2006) Host-microbe interactions: shaping the evolution of the plant immune response. Cell 124: 803–814. 16497589 10.1016/j.cell.2006.02.008

[42] Glazebrook J (2005) Contrasting mechanisms of defense against biotrophic and necrotrophic pathogens. Annu Rev Phytopathol 43: 205–227. 16078883 10.1146/annurev.phyto.43.040204.135923

[43] Leister D (2004) Tandem and segmental gene duplication and recombination in the evolution of plant disease resistance genes. Trends Genet 20: 116–122. 15049302 10.1016/j.tig.2004.01.007

[44] Chen Q, Han Z, Jiang H, Tian D, Yang S (2010) Strong positive selection drives rapid diversification of *R*-genes in Arabidopsis relatives. J Mol Evol 70: 137–148. 20044783 10.1007/s00239-009-9316-4

[45] Ding J, Zhang W, Jing Z, Chen J-Q, Tian D (2007) Unique pattern of *R*-gene variation within populations in Arabidopsis. Mol Genet Genomics 277: 619–629. 17277944 10.1007/s00438-007-0213-5

[46] Sanzol J (2010) Dating and functional characterization of duplicated genes in the apple (*Malus domestica* Borkh.) by analyzing EST data. BMC Plant Biol 10: 87. 20470375 10.1186/1471-2229-10-87 PMC3095355

[47] Lynch M, Conery JS (2000) The evolutionary fate and consequences of duplicate genes. Science 290: 1151–1155. 11073452 10.1126/science.290.5494.1151

[48] Tang H, Wang X, Bowers JE, Ming R, Alam M, et al (2008) Unraveling ancient hexaploidy through multiply-aligned angiosperm gene maps. Genome Res 18: 1944–1954. 18832442 10.1101/gr.080978.108 PMC2593578

[49] Plocik A, Layden J, Kesseli R (2004) Comparative analysis of NBS domain sequences of *NBS-LRR* disease resistance genes from sunflower, lettuce, and chicory. Mol Phylogenet Evol 31: 153–163. 15019616 10.1016/S1055-7903(03)00274-4

[50] Evans RC, Alice LA, Campbell CS, Kellogg EA, Dickinson TA (2000) The granule-bound starch synthase (GBSSI) gene in the rosaceae: multiple loci and phylogenetic utility. Mol Phylogenet Evol 17: 388–400. 11133193 10.1006/mpev.2000.0828

[51] Potter D, Gao F, Bortiri PE, Oh SH, Baggett S (2002) Phylogenetic relationships in Rosaceae inferred from chloroplast matK and trnL-trnF nucleotide sequence data. Plant Syst Evol 231: 77–89.

[52] Jordan IK, Makarova KS, Spouge JL, Wolf YI, Koonin EV (2001) Lineage-specific gene expansions in bacterial and archaeal genomes. Genome Res 11: 555–565. 11282971 10.1101/gr.166001 PMC311027

[53] Pushker R, Mira A, Rodriguez-Valera F (2004) Comparative genomics of gene-family size in closely related bacteria. Genome Biol 5: R27. 15059260 10.1186/gb-2004-5-4-r27 PMC395786

[54] Demuth JP, De Bie T, Stajich JE, Cristianini N, Hahn MW (2006) The evolution of mammalian gene families. PloS one 1: e85. 17183716 10.1371/journal.pone.0000085 PMC1762380

[55] Prachumwat A, Li W-H (2008) Gene number expansion and contraction in vertebrate genomes with respect to invertebrate genomes. Genome Res 18: 221–232. 18083775 10.1101/gr.7046608 PMC2203620

[56] Demuth JP, Hahn MW (2009) The life and death of gene families. Bioessays 31: 29–39. 19153999 10.1002/bies.080085

[57] Maere S, De Bodt S, Raes J, Casneuf T, Van Montagu M, et al (2005) Modeling gene and genome duplications in eukaryotes. Proc Nat Acad Sci USA 102: 5454–5459. 15800040 10.1073/pnas.0501102102 PMC556253

[58] Seoighe C, Gehring C (2004) Genome duplication led to highly selective expansion of the *Arabidopsis thaliana* proteome. Trends Genet 20: 461–464. 15363896 10.1016/j.tig.2004.07.008

[59] Finn RD, Mistry J, Schuster-Bockler B, Griffiths-Jones S, Hollich V, et al (2006) Pfam: clans, web tools and services. Nucleic Acids Res 34: D247–D251. 16381856 10.1093/nar/gkj149 PMC1347511

[60] Durbin R, Eddy S, Krogh A, Mitchison G (1998) Biological sequence analysis: probabilistic models of proteins and nucleic acids: probabilistic models of proteins and nucleic acids. Cambridge: Cambridge University Press. 366 p.

[61] Lupas A, Vandyke M, Stock J (1991) Predicting coiled coils from protein sequences. Science 252: 1162–1164. 2031185 10.1126/science.252.5009.1162

[62] Calenge F, Van der Linden CG, Van de Weg E, Schouten HJ, Van Arkel G, et al (2005) Resistance gene analogues identified through the NBS-profiling method map close to major genes and QTL for disease resistance in apple. Theor Appl Genet 110: 660–668. 15647920 10.1007/s00122-004-1891-6

[63] Lee SY, Seo JS, Rodriguez-Lanetty M, Lee DH (2003) Comparative analysis of superfamilies of NBS-encoding disease resistance gene analogs in cultivated and wild apple species. Mol Genet Genomics 269: 101–108. 12715158 10.1007/s00438-003-0816-4

[64] Karplus K, Barrett C, Hughey R (1998) Hidden Markov models for detecting remote protein homologies. Bioinformatics 14: 846–856. 9927713 10.1093/bioinformatics/14.10.846

[65] Felsenstein J (2006) Accuracy of coalescent likelihood estimates: do we need more sites, more sequences, or more loci? Mol Biol Evol 23: 691–700. 16364968 10.1093/molbev/msj079

[66] Zhang Z, Li J, Zhao X-Q, Wang J, Wong GK-S, et al (2006) KaKs_Calculator: calculating Ka and Ks through model selection and model averaging. Genomics Proteomics Bioinformatics 4: 259–263. 17531802 10.1016/S1672-0229(07)60007-2 PMC5054075

[67] Calenge F, Faure A, Goerre M, Gebhardt C, Van de Weg WE, et al (2004) Quantitative trait loci (QTL) analysis reveals both broad-spectrum and isolate-specific QTL for scab resistance in an apple progeny challenged with eight isolates of *Venturia inaequalis* . Phytopathology 94: 370–379. 18944113 10.1094/PHYTO.2004.94.4.370

[68] Soufflet-Freslon V, Gianfranceschi L, Patocchi A, Durel CE (2008) Inheritance studies of apple scab resistance and identification of *Rvi14*, a new major gene that acts together with other broad-spectrum QTL. Genome 51: 657–667. 18650955 10.1139/G08-046

[69] Calenge F, Durel CE (2006) Both stable and unstable QTLs for resistance to powdery mildew are detected in apple after four years of field assessments. Mol Breed 17: 329–339.

[70] Stoeckli S, Mody K, Gessler C, Patocchi A, Jermini M, et al (2008) QTL analysis for aphid resistance and growth traits in apple. Tree Genet Genomes 4: 833–847.

[71] Gardiner S, Norelli J, Silva Nd, Fazio G, Peil A, et al (2012) Putative resistance gene markers associated with quantitative trait loci for fire blight resistance in *Malus* ‘Robusta 5’ accessions. BMC Genet 13: 25. 22471693 10.1186/1471-2156-13-25 PMC3443455

[72] Le Roux PM, Khan MA, Broggini GA, Duffy B, Gessler C, et al (2010) Mapping of quantitative trait loci for fire blight resistance in the apple cultivars ‘Florina’ and ‘Nova Easygro’. Genome 53: 710–722. 20924420 10.1139/g10-047

[73] Stoeckli S, Mody K, Patocchi A, Kellerhals M, Dorn S (2009) Rust mite resistance in apple assessed by quantitative trait loci analysis. Tree Genet Genomes 5: 257–267.

[74] Soriano JM, Joshi SG, Kaauwen M, Noordijk Y, Groenwold R, et al (2009) Identification and mapping of the novel apple scab resistance gene *Vd3* . Tree Genet Genomes 5: 475–482.

[75] Galli P, Patocchi A, Broggini GA, Gessler C (2010) The *Rvi15* (*Vr2*) apple scab resistance locus contains three TIR-NBS-LRR genes. Mol Plant Microbe Interact 23: 608–617. 20367469 10.1094/MPMI-23-5-0608

[76] Bus VG, Rikkerink EH, Caffier V, Durel CE, Plummer KM (2011) Revision of the nomenclature of the differential host-pathogen interactions of *Venturia inaequalis* and *Malus* . Annu Rev Phytopathol 49: 391–413. 21599495 10.1146/annurev-phyto-072910-095339

[77] Dunemann F, Peil A, Urbanietz A, Garcia-Libreros T (2007) Mapping of the apple powdery mildew resistance gene *Pl1* and its genetic association with an NBS-LRR candidate resistance gene. Plant Breed 126: 476–481.

[78] Cevik V, King J (2002) High-resolution genetic analysis of the *Sd-1* aphid resistance locus in *Malus* spp. Theor Appl Genet 105: 346–354. 12582537 10.1007/s00122-002-0904-6

[79] Bus VGM, Chagné D, Bassett HCM, Bowatte D, Calenge F, et al (2008) Genome mapping of three major resistance genes to woolly apple aphid (*Eriosoma lanigerum* Hausm.). Tree Genet Genomes 4: 223–236.

[80] Zhang X, Feng Y, Cheng H, Tian D, Yang S, et al (2011) Relative evolutionary rates of NBS-encoding genes revealed by soybean segmental duplication. Mol Genet Genomics 285: 79–90. 21080199 10.1007/s00438-010-0587-7

