# Supplementary figures and images for: Characterization of Resistance Gene Analogues (RGAs) in Apple (Malus × domestica Borkh.) and Their Evolutionary History of the Rosaceae Family
Source: PLoS One. 2014 Feb 5;9(2):e83844. doi: 10.1371/journal.pone.0083844 (PMC3914791; doi:10.1371/journal.pone.0083844)

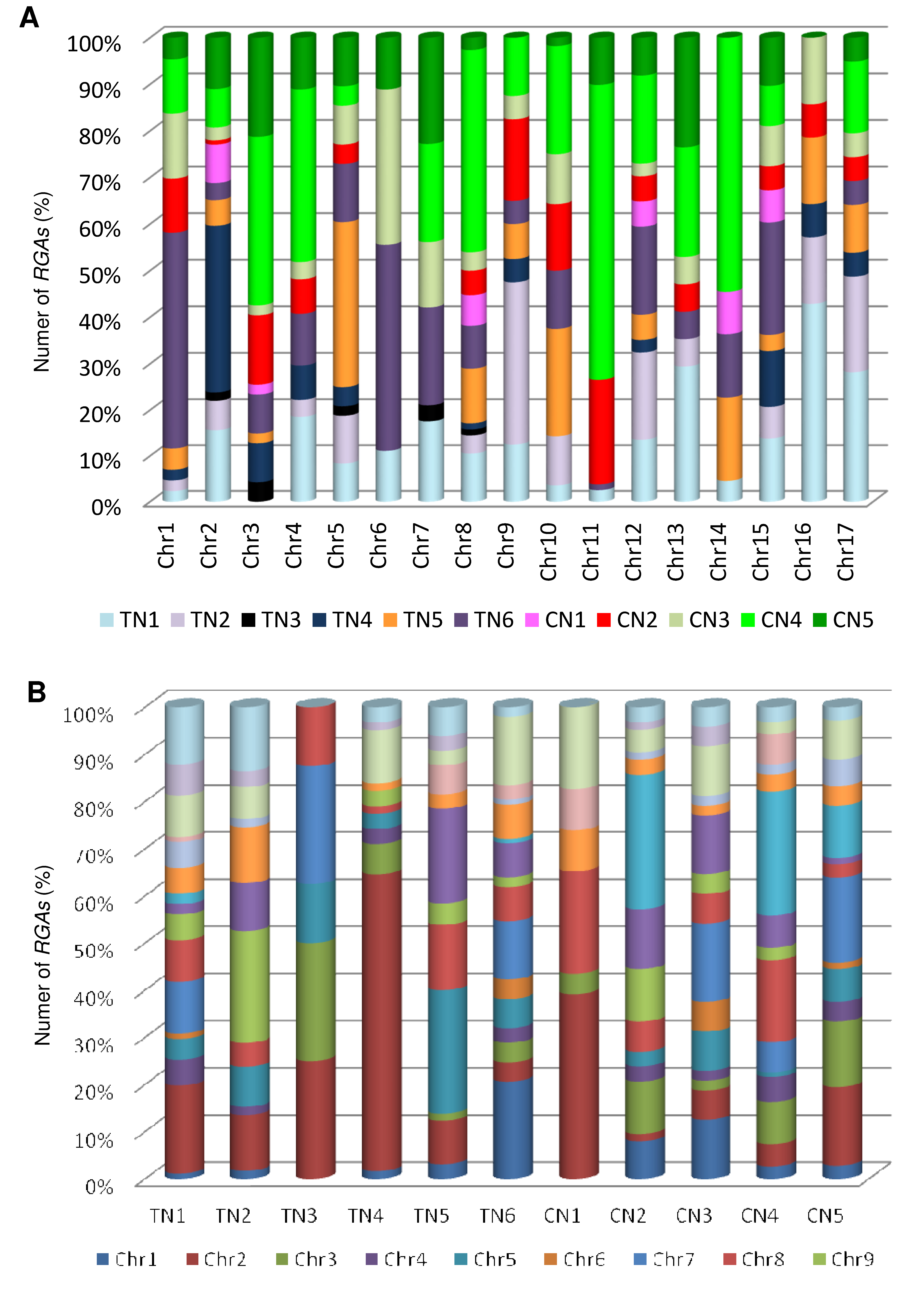

Supplement: Figure S1 — A: Distribution (percentage) of the major phylogenetic clades of apple RGAs (Figure1A) on the 17 M. domestica chromosomes (Chr). B: Percentage of chromosome (Chr) assignment to the major phylogenetic clades. Colours of major phylogenetic clades and chromosomes are listed below each chart. (TIF) [file pone.0083844.s001.tif]

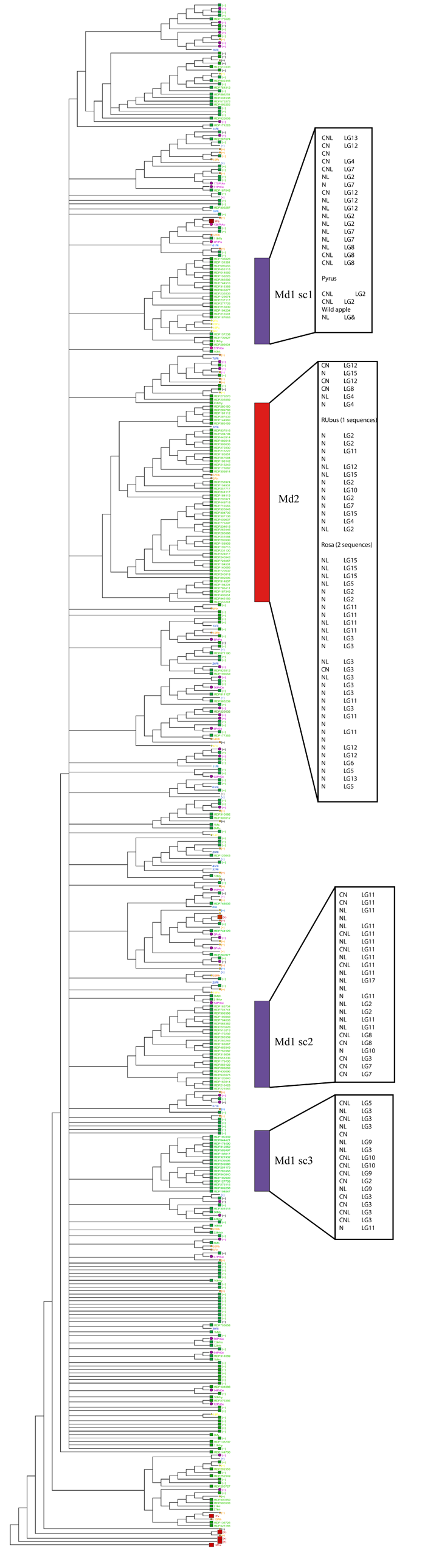

Supplement: Figure S2 — Phylogenesis of RGAs from Rosaceae species. Phylogenetic analysis of the NBS domain was carried out by the neighbor-joining method [65] using RGA sequences of domesticated and wild Malus species (green), Pyrus spp. (yellow), Prunus spp. (purple), Fragaria spp. (red), Rosa spp. (orange), and Rubus spp. (blue). The composition of the phylogenetic clades (Md1 and Md2; Figure 4) and subclades (sc) of sequences mainly from M. domestica is highlighted. Proteins present in contiguous positions on the tree are merged (collapsed branches are indicated by the + sign). (TIF) [file pone.0083844.s002.tif]

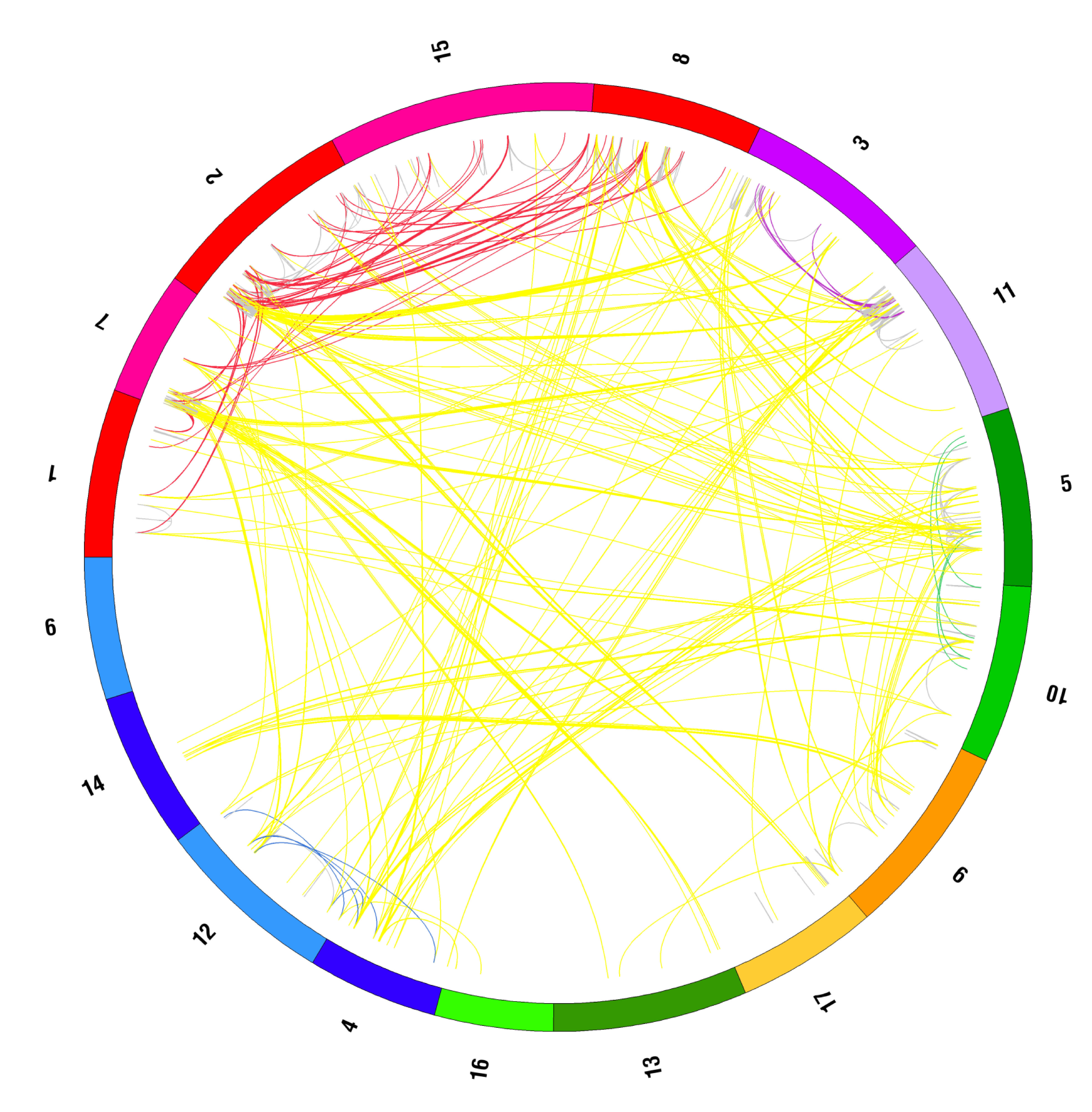

Supplement: Figure S3 — Connections between apple chromosomes based on Ks values from pairwise comparisons of RGAs . Joining lines represent connections between two RGAs among duplicated chromosomes [35] (blue, red, pink, green), among not duplicated chromosomes (yellow), and within the same chromosome (gray). Each line represents a connection between two RGAs with a Ks value lower than 0.25 [35]. A connection between two chromosomes was accepted if at least ten pairwise comparisons had a Ks value lower than 0.25. (TIF) [file pone.0083844.s003.tif]

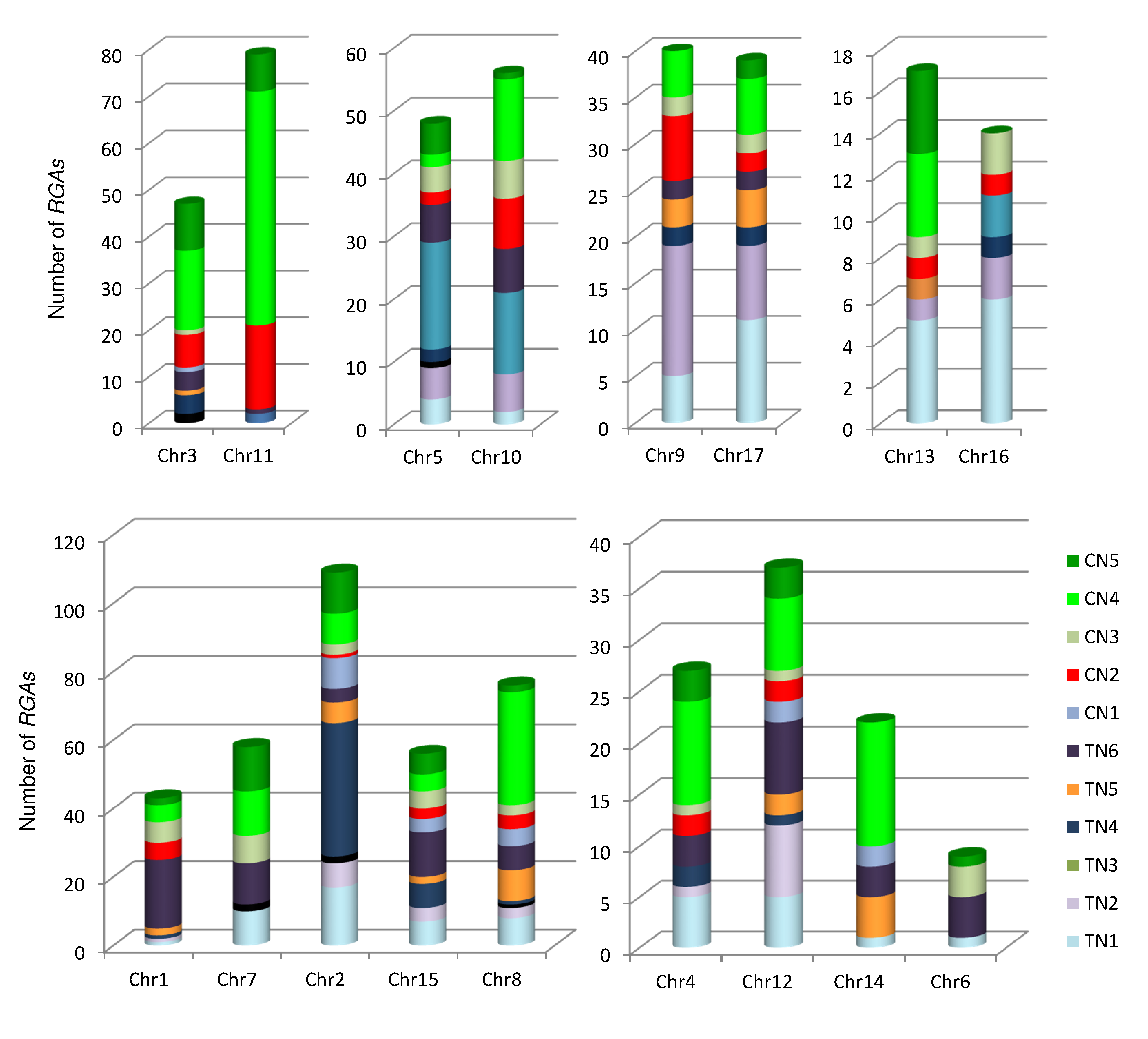

Supplement: Figure S4 — Distribution of RGAs among chromosome (Chr) doublets derived from the recent whole genome duplication of apple [34] . Colours of major phylogenetic clades (Figure 1A) are indicated. (TIF) [file pone.0083844.s004.tif]
