# Supplementary material for: Characterization of Resistance Gene Analogues (RGAs) in Apple (Malus × domestica Borkh.) and Their Evolutionary History of the Rosaceae Family
Source: PLoS One. 2014 Feb 5;9(2):e83844. doi: 10.1371/journal.pone.0083844 (PMC3914791; doi:10.1371/journal.pone.0083844)
Supplement: Table S2 — List of wild Malus species accessions (USDA apple germplasm collection at Geneva, NY, USA; www.ars-grin.gov/npgs/index.html ) used for the isolation of RGAs . (DOCX) [file pone.0083844.s006.docx]

**Table S2.**  List of wild *Malus* species accessions (USDA apple germplasm collection at Geneva, NY, USA; www.ars-grin.gov/npgs/index.html) used for the isolation of *NBS-R* genes.

| **Genus species** | **Further denomination/variety/pedigree** | **Accession ID** | **Country or region of origin/** |
| --- | --- | --- | --- |
| *Malus baccata* | Hansen's #2 | PI589838 | Former Soviet Union |
| *Malus florentina* | Skopje P2 | PI589385 | United Kingdom |
| *Malus floribunda* | 821 | PI589827 | Japan |
| *Malus fusca* |  | PI589941 | United States |
| *Malus halliana* | Parkman | PI589246 | Japan |
| *Malus honanensis* |  | PI594113 | China |
| *Malus hupehensis* | CH97 03-01 | PI633807 | China |
| *Malus kansuensis* | CH97 03-11 | PI633809 | China |
| *Malus micromalus* |  | PI589955 | UNKNOWN |
| *Malus orientalis* | 99TU-08-02 | PI633828 | Turkey |
| *Malus prattii* |  | PI588933 | UNKNOWN |
| *Malus prunifolia* | Microcarpa | PI594109 | United States |
| *Malus pumila* |  | PI594106 | China |
| *Malus sargenti* |  | PI588761 | UNKNOWN |
| *Malus sieboldii* | CH97 05-06 | PI633814 | China |
| *Malus sieversii* |  | PI596282 | Kazakhstan |
| *Malus sikkimensis* |  | PI589390 | United Kingdom |
| *Malus sylvestris* | Barenhecke 3 x Klipphausen | GMAL4497 | Germany |
| *Malus transitoria* | CH97 02-03 | PI633805 | China |
| *Malus yunnanesis* | Veitchii | PI589758 | China |
| *Malus robusta* | Robusta 5 | PI588825 | Canada |
| *Malus sublobata* | Novole | PI590174 | United States |
| *Malus hybrid* | White Angel | PI588992 | United States |
